# Physical diseases among persons with obsessive compulsive symptoms and disorder: a general population study

**DOI:** 10.1007/s00127-014-0895-z

**Published:** 2014-06-08

**Authors:** Cornelia Witthauer, Andrew T. Gloster, Andrea Hans Meyer, Roselind Lieb

**Affiliations:** Department of Psychology, Division of Clinical Psychology and Epidemiology, University of Basel, Missionsstrasse 62a, 4055 Basel, Switzerland

**Keywords:** OCD, Subthreshold types, Physical disease, Epidemiology, Obsessive compulsive symptoms, Disability

## Abstract

**Purpose:**

This study aimed at evaluating the comorbidity between DSM-IV obsessive compulsive disorder (OCD) and subthreshold forms and physical diseases in the general population as well as disability associated with comorbidity.

**Methods:**

We used data from the 1998 German Mental Health Survey, a representative survey of the German population. Mental disorders and physical diseases of 4181 subjects (aged 18–65) were cross-sectionally assessed. Mental disorders were diagnosed using the M-CIDI/DIA-X interview. Physical diseases were assessed through a self-report questionnaire and a standardized medical interview. We created three groups of obsessive–compulsive symptoms: (1) no obsessive compulsive symptoms (*n* = 3,571); (2) obsessive compulsive symptoms (OCS, *n* = 371; endorsement of OCS (either obsession or compulsion) without fulfilling any core DSM-IV criteria); (3) subthreshold OCD/OCD (*n* = 239; fulfilling either some or all of the core DSM-IV criteria).

**Results:**

In comparison to subjects without OCS, subjects with subthreshold OCD/OCD showed higher prevalence rates of migraine headaches (OR 1.7; 95 % CI 1.1–2.5) and respiratory diseases (OR 1.7; 95 % CI 1.03–2.7); subjects with OCS showed higher prevalence rates of allergies (OR 1.6; 95 % CI 1.1–2.8), migraine headaches (OR 1.9; 95 % CI 1.4–2.7) and thyroid disorders (OR 1.4; 95 % CI 1.01–2.0). Subjects with both OCS and physical disease reported the highest number of days of disability due to physical or psychological problems during the past 30 days compared to subjects with only OCS, only physical disease or neither of them.

**Conclusions:**

OCD and subthreshold forms are associated with higher comorbidity rates with specific physical diseases and higher disability than subjects without OCS. Possible etiological pathways should be evaluated in future studies and clinicians in primary care should be aware of these associations.

## Introduction

There is increasing evidence that mental disorders frequently co-occur with physical diseases [1–4]. Such comorbidity between mental and physical diseases has been found in patients diagnosed with schizophrenia, bipolar disorder, schizoaffective disorder and major depressive disorder [5]. Among these patients, nutritional and metabolic diseases, cardiovascular diseases, viral diseases, respiratory tract diseases, musculoskeletal diseases, pregnancy complications and stomatognathic diseases were found to be more prevalent than in the general population [1, 6, 7]. Due to their higher risk of cardiovascular diseases, patients with affective disorders are even known to be at high risk for premature death [4, 8].

There is evidence from patients and community-based studies that physical health problems are also associated with anxiety disorders [2, 3, 9, 10]. Significant associations between anxiety disorders and cardiac disorders, hypertension, gastrointestinal problems, genitourinary disorders and migraine have been found in patients recruited from treatment and community sources [2]. Additionally, increased rates of arthritis, asthma and ulcers were detected in patients with anxiety disorders [10]. Likewise, population surveys showed that depressive and anxiety disorders without comorbidity were associated in equal degree with physical conditions [3]. In addition, analyses revealed that the presence of an anxiety disorder was significantly associated with thyroid disease, respiratory disease, gastrointestinal disease, arthritis, migraine headaches and allergic conditions in the general population [9]. Even community samples across different countries showed that anxiety disorders occurred at higher rates in persons with heart diseases compared to those without heart disease [11]. Moreover, community analyses revealed that specific anxiety disorders are also significantly associated with medically explained pain symptoms, unexplained pain symptoms and pain disorder [12].

This mental-physical comorbidity has negative consequences for subjects’ disability in daily life. Subjects with comorbid physical and anxiety disorders are more likely to be severely disabled than subjects with either condition alone [9, 13, 14]. This may suggest that it should be ensured that subjects with mental-physical comorbidity receive enough clinical care in order to recognize and treat both disorders.

Additionally, cross-sectional analyses of the association of specific physical diseases with certain mental disorders can lead to hypotheses concerning etiological mechanisms at least in subgroups of affected subjects. For example, asthma has been found to be associated with panic disorder in many cross-sectional community-based studies [15]. These findings stimulated longitudinal studies to evaluate the role of smoking as an etiological factor in asthma and panic disorder [15]. This illustrates how hypotheses of certain etiological factors can be derived from cross-sectional associations of mental-physical comorbidity.

Based on the fact that several studies showed associations between many mental disorders and specific physical diseases, we will report for the first time the association of specific physical diseases and OCD and disability related to this comorbidity. This is important because epidemiological studies showed that across anxiety disorders obsessive compulsive disorder (OCD) was found to be the disorder with the highest estimate of the number of life years lost due to the disease in men and second highest in women behind panic disorder [16]. Increased health care utilization among individuals with OCD [17] and decreased physical wellbeing (referring to physical health, sleep and pain) in patients with OCD [18] were found. Additionally, one study revealed that the presence of any chronic physical condition increases the prevalence of obsessive–compulsive symptoms [19]. Furthermore, it is known that subthreshold types of OCD that do not fulfill all DSM-IV diagnostic criteria are more prevalent in the general population compared to OCD [20–22]. Adam et al. [17] could show that subjects with such “subthreshold” OCD (i.e. fulfilling some but not all core DSM-IV criteria) and obsessive compulsive symptoms (i.e. endorsement of stem questions without fulfilling any core DSM-IV criteria) report higher disability and increased health care utilization in the community than subjects without these symptoms.

To our knowledge no community study about the physical health problems of individuals with OCD and subthreshold forms has been published, even though subthreshold forms of OCD are known to be associated with comparable disability as full diagnostic OCD. As shown above, these analyses are relevant for implications of the health care system and to generate etiological hypotheses of OCD.

In this report we, therefore, evaluate the association between physical diseases and individuals with OCD and subthreshold forms in the general population and the disability associated with comorbidity. For this purpose we use representative community data from the German Health Interview and Examination Survey and its Mental Health Supplement.

## Method

### Design and sample

We used Data from the German Health Interview and Examination Survey and its Mental Health Supplement (GHS-MHS) conducted in 1997. The GHS was the first nationwide cross-sectional study for medical and social assessments in Germany and was commissioned by the German Ministry of Science, Research and Education and the Robert Koch Institute and authorized by the relevant institutional review board and ethics committee. The aim of the core study was the assessment of sociodemographic characteristics, physical diseases, impairments and healthcare utilization in a representative community sample of 7,124 subjects aged 18–79 (overall response rate 61.5 %). It was a stratified, randomized sample from 113 communities throughout Germany with 130 sampling units (sampling steps: (1) selection of communities, (2) selection of sampling units, (3) selection of inhabitants) [23, 24]. To handle the stratified sampling design the data were weighted and confidence intervals were calculated by the Huber-White sandwich method to account for the weighting scheme as well as the stratified sampling design [24].

For the assessment of mental disorders in the GHS-MHS, a two-stage design was used: The first stage entailed the administration of a 12-item screening questionnaire for mental disorders at the end of the medical examination of the core survey (CID-S) [25]. The second stage involved the administration of a structured psychopathological interview, the Munich Composite International Diagnostic Interview (DIA-X/M-CIDI), to all core survey subjects who had screened positive for a mental disorder and to a random sample of 50 % who screened negative [25]. This subsample of the GHS built the sample of the Mental Health Supplement and included 4,181 subjects aged 18–65 years. The conditional response rate (i.e., subjects who completed the M-CIDI interview) was 87.6 %. All subjects gave their informed consent. Further description of aims, design and methods as well as sociodemographic characteristics of the whole GHS-MHS sample can be found elsewhere [23].

### Assessment of OCD

For the diagnostic assessments, a modified version of the fully structured interview DIA-X/M-CIDI was used [26]. The questions covered DSM-IV and ICD-10 criteria. The DIA-X interview enables the assessments of symptoms, syndromes and onset, duration and severity. The interview was conducted by trained psychologists and physicians [27]. The test–retest reliability for OCD was found to be excellent (*k* = 0.81) with an average time interval of 38 days between interviews in a sample of 60 subjects in the community. The validity of the DIA-X/M-CIDI OCD diagnoses compared to diagnoses from independent treating physicians in a sample of 68 randomly chosen patients was also excellent (*k* = 0.91). The sensitivity was 100 %, while the specificity was 98.4 % [17, 28].

The DIA-X/M-CIDI module for OCD includes two parts: one for the assessment of obsessions and one for the assessment of compulsions. In each part, stem questions are asked at the beginning. In the obsession section the stem question refers to a wide range of potential thoughts and cognitions presented in the form of a symptom list: “During the last 12 month, have you been bothered by having certain unpleasant thoughts or images like recurrent arbitrary thoughts, such as the idea that your hands are dirty or have germs on them?” (yes or no). In the compulsion section three stem questions are asked to assess repetitive behaviors (“doing something like washing hands over and over again (yes or no) or “checking several times whether the door is locked” (yes or no) or mental acts (“counting something like tiles in a floor” (yes or no)). If the subject approves one of these stem questions, they are subsequently asked for the mandatory DSM-IV criteria. The DSM-IV mandatory criteria include criteria A for the diagnostic details of obsessions and compulsions, criteria B for the recognition that the obsessions or compulsions are excessive or unreasonable and C for the evaluation if the disorder causes distress or dysfunction. Diagnostic criteria refer to the past 12 months.

To facilitate comparisons with prior work [17], this paper splits the sample into the following three mutually exclusive groups:

1. Subthreshold OCD/OCD (either (a) subthreshold OCD: the subject affirmed at least one of the stem questions for obsessive or compulsive symptoms and fulfilled at least one of the DSM-IV diagnostic criteria A, B or C, but not the full DSM-IV criteria, or (b) OCD: the subject met full DSM-IV criteria A, B and C for OCD)
2. OCS (the subject affirmed at least one of the stem questions for obsessive or compulsive symptoms, but did not fulfill any of the DSM-IV criteria A, B or C)
3. No OCS (the subject did not affirm any of the stem questions for obsessions or compulsions)

Due to the small group size of full diagnostic OCD (see Table 1) and the small cell sizes when combined with physical diseases, we merged the groups OCD and subthreshold OCD (group 1) in contrast to Adam et al. [17].

**Table 1:** 12-month prevalences of no OCS, OCS and subthreshold OCD/OCD and physical diseases in the total sample (*n* = 4,181)

	No. (%)
no OCS	3,571 (86.5)
OCS	371 (8.3)
Subthreshold OCD/OCD	239 (5.2)
Subthreshold OCD	201 (4.5)
OCD	38 (0.7)
Hypertension	581 (13.1)
Cardiac diseases (heart circulation disturbances, Narrowing of the coronary vessels, angina, pectoris, cardiac infarct, heart weakness, heart, insufficiency)	100 (2.2)
Respiratory diseases (asthma, chronic bronchitis)	284 (7.0)
Gastrointestinal diseases (ulcer, gastritis)	268 (6.3)
Diabetes (with or without insulin treatment)	115 (2.7)
Arthritic conditions (wear and tear type, inflammatory diseases of the joints)	1,107 (25.9)
Allergies (hay fever, allergic eczema, allergic hives, neurodermatitis, food allergy, allergic conjunctivitis)	747 (18.1)
Migraine headaches	491 (10.3)
Neurological diseases (epilepsy, parkinson disease, multiple sclerosis)	27 (0.5)
Thyroid diseases	445 (10.0)
Vascular diseases (stroke, brain circulation disturbance, leg circulation disturbances, artery occlusion, varicose veins, vein thrombosis)	536 (12.4)

*OCS* obsessive–compulsive symptoms, *OCD* obsessive–compulsive disorder, *No.* unweighted number of subjects, *%* weighted percentage

### Assessment of physical conditions

In the GHS, physical conditions were assessed by a self-report questionnaire and a standardized computer-assisted medical interview by a general practice physician.

Based on the information on physical diseases in the self-report questionnaire, the physicians conducted information about lifetime prevalences, 12-month prevalences and point prevalences (4 weeks) of 44 physical diseases [23].

Additionally anthropometric and blood pressure measurements were conducted as well as blood and urine samples. Based on these laboratory analyses diagnoses were then supplemented and revised [23]. The following analyses are based on the physicians’ diagnoses during the medical interview. We, therefore, grouped the disorders into eleven groups of disorders (see Table 1).

### Assessment of disability

Disability was assessed by asking the subjects whether he or she was completely or partially unable to carry out daily activities (i.e., function in work, in school or in family), because of psychological problems in the 4 weeks before the interview took place (yes or no) and whether he or she was completely or partially unable to carry out daily activities (function in work, in school or in family), because of physical problems in the 4 weeks before the interview took place (yes or no).

### Sociodemographic correlates

For the present sample, earlier analyses revealed no associations between OCS, subthreshold OCD, OCD and gender, employment status and social class [17]. Significant associations between age and subthreshold OCD and OCS were found: the 12-month prevalence was lower in the older age group. Additionally, the 12-month prevalence of OCS was higher in the separated, divorced or widowed subjects group. More details can be seen elsewhere [17].

### Statistical analyses

#### Comorbidity between OCS and physical diseases

We used logistic regression [odds ratio (OR) with 95 % confidence intervals (CI)] to examine associations between the groups OCS and subthreshold OCD/OCD and physical diseases. We considered a *p* value <0.05 as statistically significant. For the logistic regression analyses we used the STATA software package, version 11.0 [29].

#### Disability

To analyze the association between OCS and physical disease on the risk of physical or psychological disability we used the two factors OCS (combining both OCS groups) and physical disease (which includes any of the physical diseases), both having two levels (yes or no). To determine whether comorbidity of OCS and physical disease were associated with an increased likelihood of past 30-day disability due to physical or psychological problems, we considered the zero inflated negative binomial model and the Hurdle model as these models account for excessive zeros (85.8 % of subjects reported 0 days of disability due to physical problems and 98.7 % of subjects reported 0 days of disability due to psychological problems). These two models led to almost identical results and fitted the data equally well (based on the Akaike information criterion, AIC). We chose the Hurdle model because we think that its underlying process is somewhat more comprehensible compared with the zero inflated negative binomial model. The hurdle model (OR with 95 % CI and incidence risk ratio (IRR) with 95 % CI) assumes negative binomial (physical disability) or Poisson (psychological disability) distributed outcomes. This model accounts not only for the excessive number of zeros observed, but also for overdispersion (i.e. the fact that the observed variability in the outcome was higher than its mean, see e.g. [30]). Hurdle models consist of two parts. In the first part, a binomial model is used to model the probability of zeros versus non-zeros. In the second part that deals with the non-zero counts and which is hence often called the “count model”, a Poisson or a negative binomial model is used (depending on whether the counts are over dispersed or not). As only non-zero values are considered in this second part, this model is zero-truncated [31].

Our model contained the two factors OCS and physical disease plus the interaction between the two. For both factors we were thus able to test (1) whether they had an impact on the probability of physical or psychological disability and (2) whether they affected the number of days of disability among those subjects who have reported at least 1 day of disability. The interaction thereby tested whether subjects having both OCS and physical disease were (1) at a particularly high risk of physical or psychological impairment and (2) if so, how strongly. For calculating the hurdle model we used the software MPlus (version 6) [32].

To check for biological interaction as described by Rothman [33], i.e. whether the combined effect of both factors OCS and physical disease is larger than the sum of the individual effects of these two factors, denoting deviation from additivity in disease risks, we additionally calculated the relative excess risk due to interaction (RERI), the attributable proportion due to interaction (AP) and the synergy index (S) [34], using the software R (version 2.14) [35]. Absence of biological interaction thereby suggests independence of OCS and physical disease as risk factors of disability.

## Results

### 12-month prevalence

The 12-month prevalence rate was 8.3 % for OCS, and 5.2 % for the combined OCD and subthreshold OCD (Table 1). Among physical diseases, the highest 12-month prevalence rate was detected for arthritic conditions (25.9 %), the lowest for neurological diseases (0.5 %).

### Associations of physical diseases and OCS, subthreshold OCD/OCD

Significantly higher prevalence rates in subjects with OCS were found for allergies (OR 1.6; 95 % CI 1.1–2.8), migraine (OR 1.9; 95 % CI 1.4–2.7) and thyroid diseases (OR 1.4; 95 % CI 1.01–2.0) compared to the no OCS group (see Table 2).

**Table 2:** Associations between 12 months physical diseases and 12-months obsessive–compulsive symptoms and disorder

Physical diseases^a^	no OCS (*n* = 3,571)	OCS (*n* = 371)		Subthreshold OCD/OCD (*n* = 239)	
	No. %	No. (%)	OR (CI)	No. (%)	OR (CI)
Hypertension					
No hypertension (*n* = 3,372)	2,868 (81.6)	303 (83.5)	1.0	201 (87.4)	1.0
Hypertension (*n* = 581)	513 (13.7)	43 (9.7)	0.7 (0.4–1.0)	25 (8.7)	0.6 (0.3–1.0)
Cardiac diseases					
No cardiac diseases (*n* = 3,982)	3,394 (95.6)	358 (97.5)	1.0	230 (95.6)	1.0
Cardiac diseases (*n* = 100)	89 (2.3)	6 (1.3)	0.6 (0.2–1.4)	5 (2.8)	1.2 (0.4–3.5)
Respiratory diseases					
No respiratory diseases (*n* = 3,728)	3,201 (89.6)	321 (87.6)	1.0	206 (85.6)	1.0
Respiratory diseases (*n* = 284)	234 (6.8)	26 (6.7)	1.0 (0.6–1.6)	24 (10.8)	1.7 (1.03–2.7)*
Gastrointestinal diseases					
No gastrointestinal diseases (*n* = 3,090)	2,264 (75.5)	263 (71.3)	1.0	163 (68.3)	1.0
Gastrointestinal diseases (*n* = 268)	215 (6.1)	29 (7.2)	1.3 (0.8–2.0)	24 (8.7)	1.6 (0.9–2.7)
Diabetes					
No diabetes (*n* = 4,022)	3,430 (96.5)	360 (97.2)	1.0	232 (97.2)	1.0
Diabetes (*n* = 115)	102 (2.8)	7 (2.2)	0.8 (0.3–1.8)	6 (2.4)	0.9 (0.3–2.5)
Arthritic conditions					
No arthritic conditions (*n* = 2,907)	2,495 (70.7)	248 (69.0)	1.0	164 (70.6)	1.0
Arthritic conditions (*n* = 1,107)	938 (25.8)	107 (27.9)	1.1 (0.8–1.5)	62 (25.1)	1.0 (0.7–1.4)
Allergies					
No allergies (*n* = 2,682)	2,334 (65.6)	207 (54.3)	1.0	141 (58.3)	1.0
Allergies (*n* = 747)	614 (17.3)	81 (23.5)	1.6 (1.1–2.8)*	52 (21.9)	1.4 (0.9–2.1)
Migraine					
No migraine (*n* = 3,497)	3,016 (86.5)	292 (80.8)	1.0	189 (80.9)	1.0
Migraine (*n* = 491)	384 (9.5)	68 (17.0)	1.9 (1.4–2.7)*	39 (14.6)	1.7 (1.1–2.5)*
Neurological diseases					
No neurological diseases (*n* = 4,114)	3,517 (99.0)	362 (98.3)	1.0	235 (98.2)	1.0
Neurological diseases (*n* = 27)	21 (0.5)	4 (0.9)	1.9 (0.6–5.8)	2 (0.9)	2.0 (0.4–9.7)
Thyroid diseases					
No thyroid diseases (*n* = 3,451)	2,973 (84.8)	284 (77.6)	1.0	194 (82.1)	1.0
Thyroid diseases (*n* = 445)	367 (9.7)	49 (12.6)	1.4 (1.01–2.0)*	29 (11.0)	1.2 (0.7–1.9)
Vascular diseases					
No vascular diseases (*n* = 3,351)	2,859 (81.1)	302 (82.8)	1.0	190 (80.8)	1.0
Vascular diseases (*n* = 536)	459 (12.5)	42 (11.2)	0.9 (0.5–1.4)	35 (13.7)	1.1 (0.7–1.7)

*OR* odds ratios from logistic regression, *CI* confidence intervals, *OCS* obsessive–compulsive symptoms, *OCD* obsessive–compulsive disorder, *No.* unweighted number of subjects, *%* weighted percentage

* *p* < 0.05

^a^Subjects reporting physical diseases during their lifetime but not within the past 12 months were excluded from the analyses

The subthreshold OCD/OCD group was associated with elevated odds for respiratory diseases (OR 1.7; 95 % CI 1.03–2.7) and migraine (OR 1.7; 95 % CI 1.1–2.5).

### Disability

#### Disability due to physical problems

We used the two factors OCS (combining both OCS groups) and physical disease (which includes any of the physical diseases) to analyze the association with disability. As there was no indication of statistical interaction between the two factors for both the binomial (*p* = 0.40) and the count model parts (*p* = 0.77), we reran the model without interaction. The binomial part of the model revealed that both OCS and physical disease significantly increased the probability of disability (OCS: OR 1.9; 95 % CI 1.4–2.5, *p* < 0.001; physical disease: OR 1.7; 95 % CI 1.2–2.2, *p* < 0.001). The count model part of the model showed that physical disease significantly increased the number of days of disability (IRR 1.6; 95 % CI 1.1–2.2, *p* = 0.008), whereas OCS did not (IRR 1.3; 95 % CI 0.9–1.8, *p* = 0.064).

**Fig. 1:**
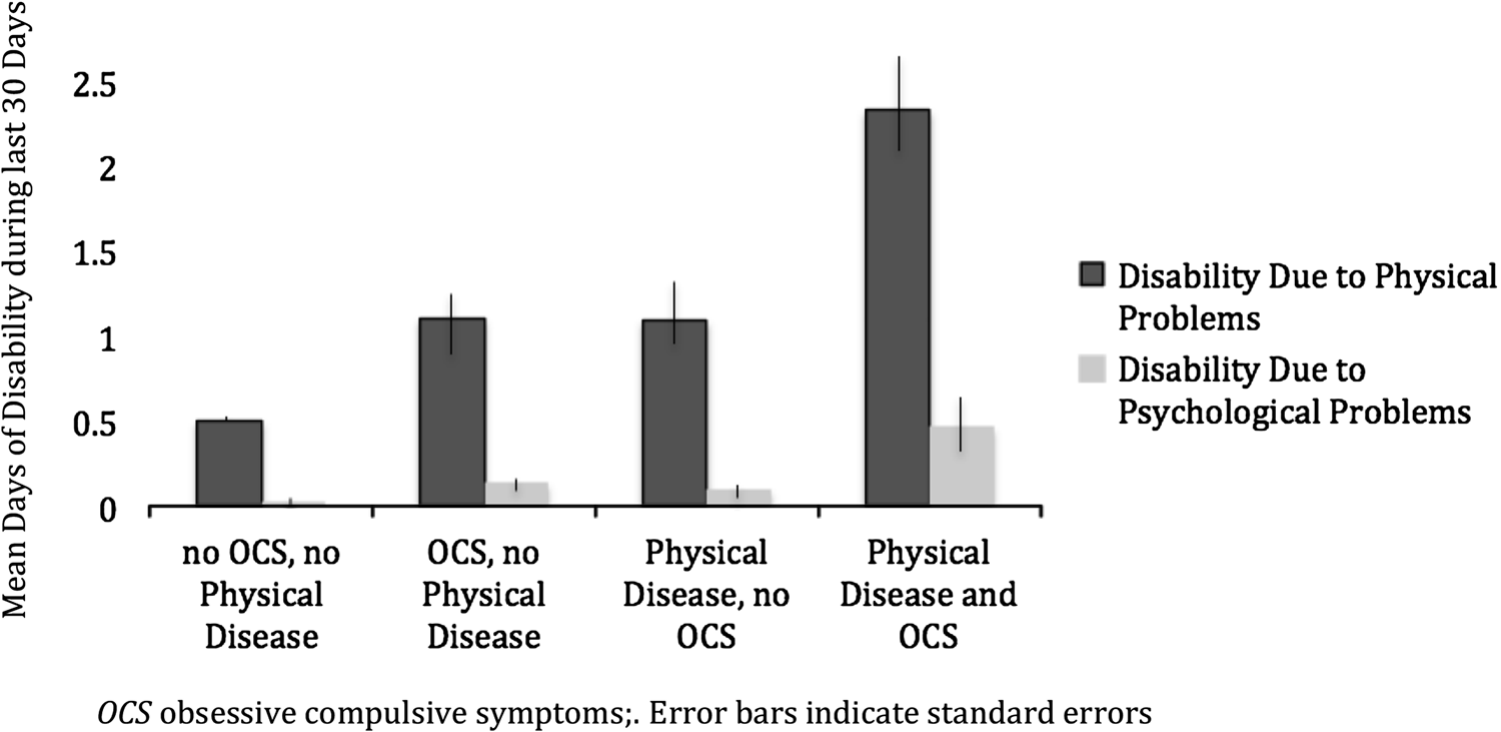
Mean days of disability during past 30 days. *OCS* obsessive compulsive symptoms; *Error bars* indicate standard errors

As shown in Fig. 1, the highest number of days of disability due to physical diseases was reported by subjects with both OCS and physical disease (*n* = 95 (2.74 %), *M* 2.33; 95 % CI 1.61–3.05), followed by subjects with OCS only (*M* 1.10; 95 % CI 0.64–1.55) and by subjects with physical disease only (*M* 1.09; 95 % CI 0.86–1.34). Subjects with neither OCS nor physical disease indicated the lowest number of days of disability (*M* 0.50; 95 % CI 0.3–0.7). There was no indication for the presence of biological interaction for any of the three measures (SI, RERI, AP; *p* > 0.05 in each case).

#### Disability due to psychological problems

As there was no indication of statistical interaction between the two factors for both the binomial (*p* = 0.24) and the count model parts (*p* = 0.74) in the disability due to psychological problems, we again reran the model without the interaction. The binomial part of the model revealed that both OCS and physical disease significantly increased the probability of disability (OCS: OR 3.8; 95 % CI 2.1–6.9, *p* < 0.01; physical disease: OR 3.7; 95 % CI 1.4–9.8, *p* < 0.01). Contrary to the disability due to physical problems, the count model part of the model revealed that there was no significant effect of both factors on the number of days of disability due to psychological problems (*p* > 0.55 for both model parts).

As for disability due to physical problems, the highest number of days of disability due to psychological problems was reported by subjects with both OCS and physical disease (*n* = 19 (0.55 %), *M* 0.47; 95 % CI 0.17–0.76), followed by subjects with OCS only (*M* 0.14; 95 % CI 0.03–0.31) and by subjects with physical disease only (*M* 0.10; 95 % CI 0.05–0.16). Subjects with neither OCS nor physical disease indicated the lowest number of disability days (*M* 0.03; 95 % CI 0.00–0.06; Fig. 1). Again, no indication for biological interaction was found for any of the three measures (SI, RERI, AP; *p* > 0.05 in each case).

## Discussion

To the best of our knowledge, this is the first study that analyses the association between OCD and subthreshold forms and physical diseases in a representative community sample.

Our results show that obsessive compulsive symptoms are associated with higher prevalence rates of specific physical diseases in the general population. These results add to the body of literature on the comorbidity of physical diseases and other anxiety disorders. In comparison to findings from other anxiety disorders, we found associations with migraine headaches, allergies and thyroid diseases in OCS and significant associations with respiratory diseases and migraine headaches in subthreshold OCD/OCD [2, 9, 10]. Further, our analyses revealed that subjects with both OCS and physical disease report the highest number of days of disability compared to subjects having only OCS (without physical disease), only a physical disease or neither of them.

Different models exist to explain the cooccurrence of anxiety disorders and physical diseases: anxiety as consequent or antecedent factor of a physical disease, third variables that lead to the comorbidity or, common genetic, environmental or personality factors that contribute to the cooccurrence [9]. Even though only few specific hints for the explanations of associations between OCS and physical diseases exist, these hints can point towards important etiological pathways in subgroups of OCD patients and, therefore, will be discussed in the following.

First, defects in serotonin metabolism as possible neurochemical basis of both migraine and OCD have been proposed [36]. An abnormal serotonin function in subjects with OCD is one of the most consistent pathophysiologic findings [37]. Similarly, serotonin abnormalities have been implicated in the pathogenesis of migraine [36, 38]. Alternatively it has been proposed that anxiety disorders may be involved in peripheral and central mechanisms of pain sensitization which contributes to the evolution of chronic headaches [39]. Against this background, our results can support a suggested role of serotonin in an etiological pathway of OCD.

Second, an increased rate of immune-related symptoms among OCD patients has been reported [40]. As a possible explanation, one theory suggests that postinfectious autoimmune responses might be associated with the development of pediatric OCD, which leads to an increased rate of immune-related diseases in adults with OCD [40]. Our cross-sectional results match with this theory, as we found increased rates of allergies in subjects with OCS. Studies showed that especially immune responses to streptococcal infections may be relevant for the etiology of OCD [41]. Our analyses support a suggested involvement of immune responses that may be relevant in the etiology of a subgroup of OCD.

Third, the association between asthma and anxiety disorders, especially in panic disorder, is well established [42, 43]. Explanations range from hyperventilation that is commonly associated with anxiety disorders, subjective psychological disturbance which could lead to enhanced bronchoconstriction to biological effects of anxiety on immunological or biological factors [44]. Specific explanations of the association between OCD and respiratory diseases lack, however. Therefore, we can only speculate that comparable to other anxiety disorders, subjects with OCD could have an altered symptom perception leading to enhanced awareness of breathlessness and bronchoconstriction and therefore to asthma-like symptoms. Despite that no specific explanation for this association exists, our results nevertheless show that an involvement of the respiratory tract may be important in OCD.

Fourth, pervasive evidence documents the relationship between thyroid diseases and mental symptoms such as impairment of cognitive functions or behavioral and mood disturbances [45, 46]. Concerning OCD, some observations of increased rates of obsessive–compulsive symptoms in subjects with thyroid disease have been found [46]. As an explanation, common biochemical abnormalities that play a role for both thyroid diseases and OCD may exist [46]. A diminished thyrotropin releasing hormone (TRH) response to a TRH stimulation was detected in subjects with OCD, also [47] suggesting an alteration in the serotonergic system, as a decreased central serotonergic activity is associated with blunted TSH response [47]. The increased rates of thyroid diseases in our analyses support the hypothesis of an alteration of the hypothalamic-pituitary-thyroid axis in the pathophysiology of OCD, too.

Fifth, it could be suggested that certain behaviors that occur in OCD increase the vulnerability to develop a physical disease. As a lack of exercise has been associated with anxiety disorders [48] and physical inactivity is associated with many chronic physical diseases such as cardiovascular diseases or diabetes [49], it could be suggested that subjects with OCD are at increased risk to develop physical illnesses through physical inactivity. Further, it has been shown that cleaning activities related to exposure to certain cleaning products in the household are associated with asthma [50]. Extensive hand washing or cleaning can be a symptom of OCD. Through exposure to poisonous cleaning agents this could lead to higher prevalence of respiratory diseases in subjects with OCD. Additionally, subjects with OCD avoid uncertainty [51]. This might in addition be related to an increased prevalence of physical diseases in these subjects, as isolation and, therefore, a lack of exercise may be the consequences.

Given these etiological considerations, our results may be useful to deduce hypotheses concerning the involvement of certain physiological factors in the etiology of OCD in subgroups of subjects. Future studies are clearly needed to replicate these findings.

As some physical diseases were only associated with OCS and not OCD (higher prevalence of allergies and thyroid diseases only in OCS) or vice versa, it is possible that different etiological factors are related to OCS, subthreshold OCD and OCD. Future studies should, therefore, not only include OCD but also subthreshold forms to test these hypotheses.

Besides the documentation of the associations between physical diseases and OCS and the deduction of potential etiological hypotheses, our analyses show that both subjects with OCS or physical diseases have an increased probability of disability due to psychological or physical problems during the past 30 days. Comparable to previous studies on anxiety disorders [9] or mental disorders in general [13] and physical diseases, the highest number of days of disability was reported in the group with both OCS and physical diseases. This is supported by previous findings that subjects with both mental and physical conditions are more likely to be severely disabled than those with either condition alone [13].

Due to the fact that no indication for biological interaction was found between OCS and physical disease, the increased disability of subjects affected by both OCS and physical disease may be seen as an additive rather than a synergistic effect of both disorders.

The mechanisms leading to this specific increased disability are unknown. Research on other anxiety disorders, however, suggest that anxiety is associated with poor adherence to self-care regimen and increased medical complications in patients with chronic medical illness [52]. This could lead to decreased active behavioral self-management strategies and, therefore, to an increased burden of the physical disease in anxiety in general [53] and specifically in OCD.

The increased disability in subjects with both OCS and physical disease may reflect an increased need of recognition and treatment of both physical disorder and OCS in primary health care. Future studies could additionally investigate whether this comorbidity is associated with a loss in quality of life.

The current study has a number of limitations. First, the survey is limited to subjects aged 18–65 years, which does not enable generalization of the results to younger or older subjects. Second, as already mentioned by Sareen et al. [9], even though physicians’ diagnoses were used, certain diagnoses are more reliant on self-report data (e.g. arthritis) than others (e.g. diabetes). That may have led to over reporting of physical symptoms in anxious patients. Third, due to the cross-sectional nature of the study, it is not possible to draw conclusions about the causal nature of the associations between OCD or OCS and physical diseases. Fourth, although we used a large representative sample with 4,181 subjects, the sample size of full diagnostic OCD is rather small (*n* = 38). In addition, the combinations between OCD symptoms and physical diseases led to small cell sizes (especially in cardiac diseases, diabetes and neurological diseases). Fifth, it has to be considered that comorbidity between OCD and subthreshold forms and other mental disorders has been reported [17]. Thus, further investigation is needed to examine specificity of the results.

With these limitations in mind, our community study shows that subjects affected by DSM-IV obsessive compulsive disorder either on the full/subthreshold or even on the symptomatic level report higher rates of certain physical diseases than subjects without these symptoms. This comorbidity is associated with higher impairment than either condition alone.

These findings can be helpful to detect new etiological pathways underlying OCD in subgroups of affected subjects or support the ones suggested in earlier studies. In addition, clinicians and doctors in primary care need to be sensibilized for these associations to recognize and treat both physical disease and OCS to reduce disability in affected subjects.

## Abbreviations

OCD: Obsessive compulsive disorder
OCS: Obsessive compulsive symptoms
GHS-MHS: German health interview and examination survey and its mental health supplement
DIA-X/M-CIDI: The Munich composite international diagnostic interview
DSM-IV: Diagnostic and statistical manual of mental disorders, Fourth Edition
ICD-10: International classification of diseases, Tenth Edition
CI: Confidence interval
OR: Odds ratio
IRR: Incidence risk ratio
RERI: Relative excess risk due to interaction
AP: The attributable proportion due to interaction
S: Synergy index
